# [Retracted] Regulation of lncRNA PVT1 on miR-125 in metastasis of gastric cancer cells

**DOI:** 10.3892/ol.2025.15209

**Published:** 2025-07-28

**Authors:** Jiatai Niu, Xiangxin Song, Xiaomei Zhang

Oncol Lett 19: 1261–1266, 2020; DOI: 10.3892/ol.2019.11195

Following the publication of the above paper, it was drawn to the Editor's attention by a concerned reader that the Transwell assay data shown in Fig. 4 on p. 1265 had already appeared in an article written by different authors at different research institutes that was published previously in the journal *Oncotarget*.

Owing to the fact that the contentious data in the above article had already been published prior to its submission to *Oncology Letters*, the Editor has decided that this paper should be retracted from the Journal. The authors were asked for an explanation to account for these concerns, but the Editorial Office did not receive a reply. The Editor apologizes to the readership for any inconvenience caused.

